# YY1 regulates melanoma tumorigenesis through a miR-9 ~ RYBP axis

**DOI:** 10.1186/s13046-015-0177-y

**Published:** 2015-06-24

**Authors:** Guowei Zhao, Qiang Li, Aiqin Wang, Jian Jiao

**Affiliations:** Department of Dermatology, The Central Hospital of Zibo City, Zibo, 255036 Shandong Province China; Oncology Department, The Foruth People’s Hospital of Zibo City, Zibo, 255067 Shandong Province China; Department of Dermatology, Qilu Hospital Shandong University, 107 Wenhuaxi Road, Jinan, 250012 Shandong Province China

**Keywords:** YY1, Melanoma tumorigenesis, miR-9 ~ RYBP axis

## Abstract

**Background:**

The Yin Yang 1 (YY1) transcription factor has been identified to target a plethora of potential target genes, which are important for cell proliferation and differentiation. Although the role that YY1 plays in different human types of cancer has been reported, its biological and mechanistic significance in melanoma has not been well defined.

**Methods:**

Quantitative RT-PCR analysis was used to determine whether aberrant YY1 and miR-9 expression occurred in melanoma, compared with benign nevi and normal tissue controls. Furthermore, the transcriptional regulation of YY1 on miR-9 expression was assessed by using quantitative ChIP-PCR assay. Subsequently, the effects of YY1 and miR-9 on proliferation, cell cycle, migration and invasion of melanoma cells were detected using CCK-8, flow cytometric analysis, wound healing and transwell invasion assays, respectively. Finally, the post-transcriptional regulation of miR-9 on RYBP was analyzed using luciferase reporter and immunoblot analysis.

**Results:**

Elevated YY1 levels were observed in patients with melanoma, compared with benign nevi and normal tissue controls, and the increased YY1 was associated with melanoma metastasis state and tumor stage. Furthermore, YY1 negatively regulated miR-9 transcription. Silencing of YY1 inhibited proliferation, cell cycle progression, migration and invasion in melanoma cells, while ectopic of miR-9 did the same. Additionally, RYBP was shown to be a direct target of miR-9 through binding to its 3′ UTR, thus forming a YY1 ~ miR-9 ~ RYBP axis.

**Conclusions:**

These results identify a novel YY1 ~ miR-9 ~ RYBP axis involved in melanoma tumorigenesis and reinforce the idea that regulatory circuitries involving miRNAs and TFs are prevalent mechanisms.

## Introduction

Yin Yang 1 (YY1), also known as δ, NF-E1, UCRBP and CF1, is a ubiquitously distributed transcription factor belonging to the GLI-Kruppel class of zinc finger proteins that is involved in repressing and activating a diverse number of promoters. Studies have demonstrated the association and modulation of YY1 by the adenovirus-derived E1A, an oncoprotein that induces YY1-mediated activation of transcription. In the absence of E1A, the activity of YY1 is reversed, converting to a transcriptional repressor, hence the name Yin Yang 1 [1–3]. Since YY1 is a general transcription factor involved in approximately 10 % of the total mammalian genes, the expression levels of YY1 must be tightly monitored for the survival of cells and organisms. Accordingly, abnormal YY1 protein levels have been shown to affect the clinical behavior of several cancer types [4–9].

The putative role of YY1 in tumorigenesis has been supported by its interaction with different molecules. Cicatiello et al. reported that cyclin D1 gene promoter activation in estrogen-responsive human breast cancer is marked by release of the YY1 transcriptional repressor complex including HDAC-1 [10]. Alternatively, YY1 has also been shown to activate c-myc promoter in tumor cells [11, 12]. Moreover, YY1 has been found to be associated with the tumor suppressor p53 [13, 14]. Sui et al. demonstrated that ablation of endogenous YY1 results in p53 accumulation due to a reduction in p53 ubiquitination in vivo [15]. Recent study has revealed that YY1 could help Polycomb Group (PcG) protein recruitment to DNA, a novel mechanism by which YY1 used to regulate tumorigenesis [16, 17].

Studies have repeatedly showed that in the majority of the tumors studied, YY1 transcript levels are significantly higher than in the relative normal counterparts for each cancer type analyzed. Varambally et al. showed that YY1 was significantly higher in primary prostate cancers than in metastatic prostate cancers [18]. YY1 was also overexpressed in colon cancer in the absence of gene amplification and chromosomal translocation [19]. Accordingly, elevated YY1 level were consecutively found in ovarian cancer, breast cancer, cervical cancer, and osteosarcoma [20]. However, there is limited information available as to the involvement of YY1 in melanoma.

Melanoma is the deadliest form of skin cancer, characterized by a rapid progression, metastasis to regional lymph nodes and distant organs as well as a limited efficiency of therapeutics [21–23]. It is readily curable if diagnosed at an early stage, however a large percentage of melanomas arise without association with premalignant nevi. This leads to ineffective early detection and results in approximately 10 % of patients presenting with metastatic disease upon first diagnosis. Although melanoma is among the most notoriously aggressive and treatment-resistant of human cancers, recent studies have yielded the identification of multiple melanoma oncogenes, several of which seem to have been successfully targeted with small molecules. For example, approximately 50 % of malignant melanomas harbor a BRAF activating mutation, the majority of these being BRAFV600E [24, 25], which results in constitutive activation of BRAF and increased activation of the MAPK pathway. Based on these findings, many preclinical studies have investigated the potential therapeutic value of targeting BRAF^V600E^, and several selective BRAF inhibitors have been developed for clinical applications in melanoma, as PLX4032 (also known as vemurafenib) [26–28], GSK2118436 (also known as dabrafenib) [29], and GSK1120212 (also known as trametinib) [30, 31]. Thus, it is essential to identify additional regulators and molecules that play critical roles in melanoma growth and progression, which could serve as the potential points of intervention for future therapies.

Given the essential nature of YY1 function in different human carcinomas, we decided to investigate the role for YY1 in human melanoma. In this study, we perform RT-qPCR analysis to detect the YY1 mRNA level in both benign nevi and melanoma specimens. Elevated YY1 levels were observed in patients with melanoma, compared with the levels detected in age/gender-matched controls with benign nevi and normal tissue controls. We further identified YY1-targeted miR-9, which was repressed in melanoma cells, was regulated by YY1 and elucidated its functional significance in regulating melanoma growth and progression. Additionally, RYBP, a PcG protein physically interacts with YY1, was identified as a novel target of miR-9. We also showed that YY1 could affect RYBP level in melanoma cells, thus forming a YY1 ~ miR-9 ~ RYBP regulatory axis. Collectively, our results may identify YY1 as a novel regulator of melanoma that modulates the miR-9 ~ RYBP axis to promote melanoma tumorigenesis.

## Materials and methods

### Human melanoma and benign nevi tissues

Paraffin-embedded pathological samples were obtained from patients who underwent surgery at Cancer Hospital of Chinese Academy of Medical Sciences from 2008 to 2014. All specimens were re-evaluated by an expert pathologist. Tissues were obtained according to local ethical guidelines and approved by the local ethics committee. Skin tissues from 12 patients with melanocytic nevus (matched by gender and age) were collected as controls. All samples were snap-frozen in liquid nitrogen, then stored at −80 °C for further use.

### Cell cultures and cell transfection

The melanoma cells lines used in this study were purchased from the Cell Resource Center of IBMS, CAMS. WM852, WM1791C and WM8 were grown in RPMI media supplemented with 10 % FBS and penicillin/streptomycin. FO-1, WM983A, WM793, Daju and WM209 were grown in MCDB135 media supplemented with 2 % FBS, insulin, CaCl2, and penicillin/streptomycin. All cells were cultured in a 5 % CO2 humidified incubator. The melanoma cell lines WM1791C and WM209 were transfected with miRNA mimic, negative mimic control, si-YY1, siRNA control (Scramble; GenePharma; Shanghai, China) at a final concentration of 25 nmol/L using DharmaFECT 1 (Dharmacon; USA) in accordance with the manufacturer’s instructions.

### RNA extraction and quantitative real-time PCR analysis (RT-qPCR)

The RNeasy FFPE Kit (Qiagen, CA, USA) was used to purify total RNA from the paraffin-embedded melanoma tissues as the manufacturer’s instructions. Total RNA was extracted from the cells and benign nevi tissues using Trizol reagent (Invitrogen, CA, USA), according to the manufacturer’s instructions. RT-qPCR assay was conducted to detect the level of YY1 mRNA and miR-9. Briefly, cDNA was synthesised by M-MLV reverse transcriptase (Invitrogen) from 5 ug of total RNA. Stem-poop RT primer was used for the reverse transcription of miR-9. RT-qPCR was performed on the Bio-rad CFX96 real-time PCR System (Bio-rad, Foster City, CA, USA) using KAPA PROBE FAST qPCR Kits (Kapa Biosystems, MA, USA) with the following cycling conditions: 95 °C for 10 min (initial denature); then 40 cycles of 95 °C for 15 s, 60 °C for 60 s. The cycle passing threshold (Ct) was recorded for each candidate miRNAs, and miR-9 quantification data were normalized to U6, YY1 quantification data were normalized to GAPDH.

### Cell proliferation assay

Cells were incubated in 10 % CCK-8 (DOJINDO, Japan) diluted in normal culture medium at 37 °C until visual color conversion occurred. Proliferation rates were determined at 0, 12, 24, 48, 72, 96 h after transfection. The absorbance of each well was measured with a microplate reader set at 450nM and 630nM. All experiments were performed in quadruplicate.

### Cell cycle analysis

WM1791C and WM209 were removed with PBS/EDTA and/or trypsin solution, and centrifuged at 1200 rpm at 4 °C for 5 min. Decant the supernatant and gently re-suspend the cells in PBS. Count cells by hemocytometer and wash one time by putting 1X10^6^ cells per tube, adding 1 ml of PBS and centrifuging at 1200 rpm at 4 °C. Re-suspend pelleted cells in 0.3 ml of PBS buffer and add 0.7 ml cold ethanol (70 %) dropwise to tube to fix the cells. Leave on ice for 1 h (or up to a few days at 4 °C), and centrifuge cells as above, wash 1 time with cold PBS and re-centrifuge. Add 10 μl of 1 mg/ml PI solution (the final concentration being 10 μg/ml) and 5 μl of 10 mg/ml Rnase A (the final concentration being 0.2 mg/ml). Keep in the dark and at 4 °C until analysis. Analyze on FACS by reading on cytometer at 488 nm.

### Cell migration and invasion assays

WM1791C and WM209 cells were grown to confluence on 12-well plastic dishes and treated with miRNA mimics or siRNAs. Then 24 h after transfection, linear scratch wounds (in triplicate) were created on the confluent cell monolayers using a 200 μL pipette tip. To remove cells from the cell cycle prior to wounding, cells were maintained in serum-free medium. To visualize migrated cells and wound healing, images were taken at 0, 12, 24, 48 h. A total of ten areas were selected randomly from each well and the cells in three wells of each group were quantified.

For the invasion assays, after 24 h transfection, 1 × 10^5^ WM1791C cells in serum-free media were seeded onto the transwell migration chambers (8 μm pore size; Millipore, Switzerland) which coated with the upper chamber of an insert coated with Matrigel (Sigma-Aldrich, USA). Media containing 20 % FBS were added to the lower chamber. After 24 h, the noninvading cells were removed with cotton wool, Invasive cells located on the lower surface of the chamber were stained with May-Grunwald-Giemsa stain (Sigma-Aldrich, USA) and counted using a microscope (Olympus, Tokyo, Japan). Experiments were independently repeated three times.

### Immunoblotting

Immunoblotting analysis was carried out using standard methods. Proteins were separated by 10 % SDS-PAGE, and transferred onto PVDF membranes (Millipore Corporation, Billerica MA, USA). Membranes were blocked overnight with 5 % non-fat dried milk for 2 h and incubated with anti-YY1 antibody (Bioworld) at 1:2000 dilution; anti-RYBP (Abcam) antibody at 1:1000 dilution, anti-GAPDH antibody (Proteintech) at 1:50,000 dilution overnight at 4 °C. After washing with TBST (10 mM Tris, pH 8.0, 150 mM NaCl, and 0.1 % Tween20), the membranes were incubated for 2 h at room temperature with goat anti-rabbit antibody (Zsgb-bio, Beijing, China) at 1:20000.

### Statistical analyses

The *t*-test and a non-parametric test were undertaken to analyze the data. A two-sided *P*-value of less than 0.05 was considered statistically significant. All statistical computations were performed using SPSS (SPSS Inc., Chicago, IL, USA).

## Results

### YY1 is frequently upregulated in melanoma tissues and cell lines

**Fig. 1:**
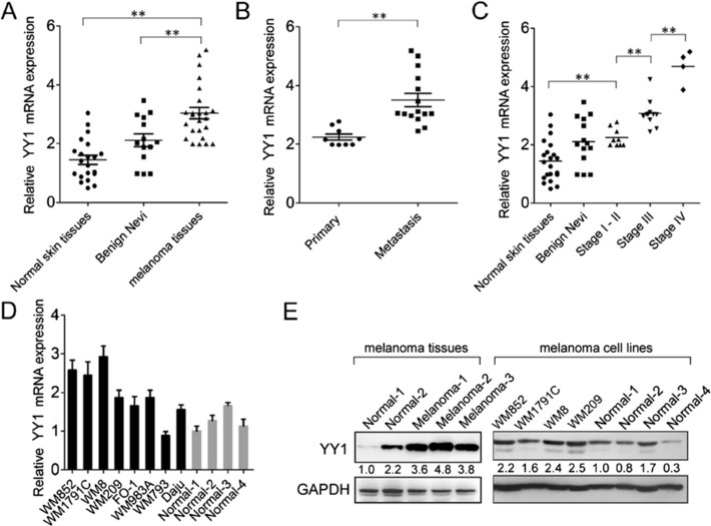
The expression analysis of YY1 in normal skin tissues, benign nevi and melanoma tissues. (**a**) Relative YY1 mRNA expression levels in normal skin tissues, benign nevi and melanoma tissues; (**b**) The Statistical analysis of the association between YY1 level and metastasis state (primary and metastasis); (**c**) The Statistical analysis of the association between YY1 level and tumor stage (I, II, III and IV); (**d**) The relative level of YY1 mRNA in melanoma cell lines (WM852, WM1791C, WM8, WM209, FO-1, WM983A, WM793 and Daju) relative to 4 normal tissue controls; (**e**) Immunoblot of endogenous YAP1 protein level in 3 melanoma tissues, GC cell lines (WM852, WM1791C, WM8 and WM209) relative to 4 normal tissue controls; An unrelated protein GAPDH was used as the control. For the quantitative results, the data are presented as the mean ± SEM, and the error bars represent the standard deviation obtained from three independent experiments. *, *p* < 0.05; **, *p* < 0.01

Given the fact that, elevated expression or nuclear enrichment of YAP has been observed in multiple types of human cancers, we first assessed the expression level of YY1 mRNA in 14 benign nevi and 24 melanoma specimens, including 15 metastatic melanoma (Fig. 1a) by RT-qPCR analysis. Normal skin tissues were used as control. As shown in Fig. 1a, a significantly increased level of YY1 was seen in patients with melanoma (primary and metastatic melanomas), compared with the levels detected in age/gender-matched controls with benign nevi (*p* < 0.01) and normal tissue controls (*p* < 0.001). Additionally, the YY1 level was observed to be higher, within tumor cells, in metastatic melanomas than in primary melanoma (*p* < 0.001) (Fig. 1b).

Melanoma patients were then grouped by clinical cancer stages. There were still positive correlations between YY1 expression and different tumor stages (*p* < 0.01, Stage I-II vs. Normal; *p* < 0.01, Stage III vs. Stage I-II; *p* < 0.001 Stage IV vs. Stage III) (Fig. 1c). However, no correlation was observed between YAP1 expression and age, gender, localization or tumor size (data not shown). We next extended our test to eight human melanoma cell lines. Levels of YY1 transcripts were highly variable across the panel. Noticeably, expression of YY1 was relatively higher in four melanoma cell lines without BRAF mutation (WM852, WM1791C, WM8 and WM209) compared with normal human melanocyte cultures (Fig. 1d). Accordingly, these cell lines showed a notable overexpression of YY1 protein compared to normal clusters with a BRAF^V600E^ mutation (Fig. 1e). The up-regulation of YY1 protein in melanoma tissues was also validated in selected samples (Fig. 1e). Taken together, these results suggest that YY1 might play a regulatory role in melanoma cell growth and migration, especially for the melanomas that do not harbor a BRAF^V600E^ mutation.

### Knockdown of YY1 inhibits melanoma cell proliferation and migration

**Fig. 2:**
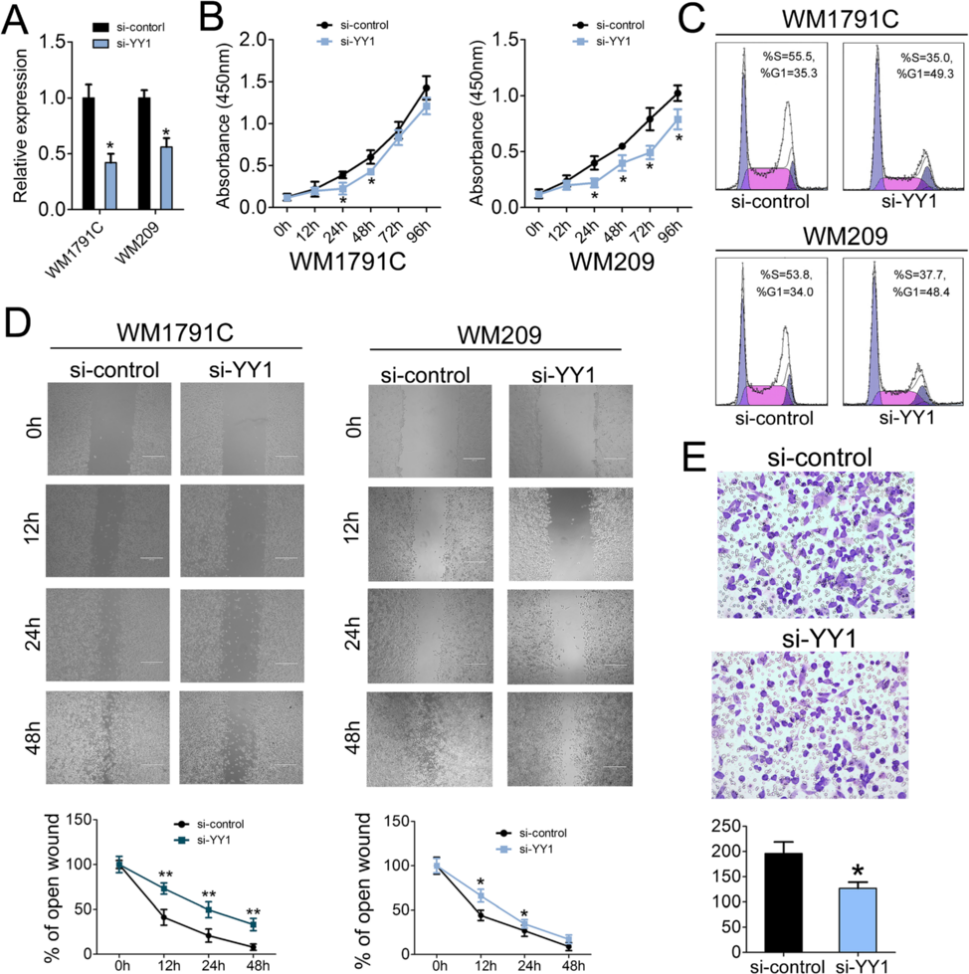
Knockdown of YY1 in melanoma cells inhibits cell proliferation, migration and invasion. (**a**) YY1 level were detected in WM1791C and WM209 cells after treatment with siRNAs specific to YY1 (25 nM) or siRNA control (25 nM) by RT-qPCR; (**b**) Cell proliferation assay of WM1791C and WM209 cells after treatment with si-YY1 or si-control by using CCK-8; (**c**) Cell cycle analysis by propidium iodide (PI) staining of WM1791C and WM209 cells after treatment with si-YY1 or si-control; (**d**) Wound healing assays of WM1791C (the left panel) and WM209 (the right panel) cells after treatment with si-YY1 or si-control, The relative ratio of wound closure per field was shown in the below; (**e**) Transwell analysis of WM1791C cells after treatment with si-YY1 or si-control, The relative ratio of invasive cells per field was shown below. For the quantitative results, the data are presented as the mean ± SEM, and the error bars represent the standard deviation obtained from three independent experiments. *, *p* < 0.05; **, *p* < 0.01

Because the YY1 was found to be expressed at higher levels in melanoma cell lines WM852, WM1791C, WM8 and WM209, we chose WM1791C and WM209 to perform further assays to test whether YY1 was functionally involved in malignant melanoma tumorigenesis. siRNAs specific to YY1 were transfected into the WM1791C and WM209 cells and the level of YY1 was subsequently confirmed by RT-qPCR analysis (Fig. 2a). siRNA transfected cells exhibited significantly decreased proliferation compared with control siRNA transfected WM1791C and WM209 cells (Fig. 2b). Accordingly, the percentage of S phase cells was also reduced by ~20 % and ~18 % in si-YY1 transfected WM1791C and WM209 cells respectively (Fig. 2c). Taken together, these results indicated that inhibition of YY1 can efficiently inhibited tumor cell proliferation and cell cycle in vitro, suggesting its oncogenic role in modulating tumorigenicity of melanoma cells.

The rapid invasion and metastasis of tumor cells are responsible for poor prognosis and the major cause of death in melanoma patients. Based on above statistic results that YY1 expression displayed close association with metastasis and malignant degree of tumor, we proposed that it might play an extremely important role in melanoma cell migration and invasion. To test our hypothesis, cell migration and invasion assays were performed in WM1791C and WM209 cells transfected with either si-YY1 or siRNA controls (Fig. 2a). Therefore, the inhibition of endogenous YY1 resulted in a significant reduction of cell migration during the closing of an artificial wound created over a confluent monolayer (Fig. 2d). Moreover, these cells were maintained in serum-free medium during the course of wound healing to ensure that any augmented migratory behavior could not be affected by altered cell proliferation. In addition, reduced expression of YY1 dramatically inhibited the normally strong invasive capacity of WM1791C cells as indicated in the transwell invasion assay (Fig. 2e). These results were consistent with the above findings that the YY1 could promote tumor cells growth as well as their progression towards more malignant degree.

### YY1 resided on the upstream of miR-9 loci and inhibited its expression

**Fig. 3:**
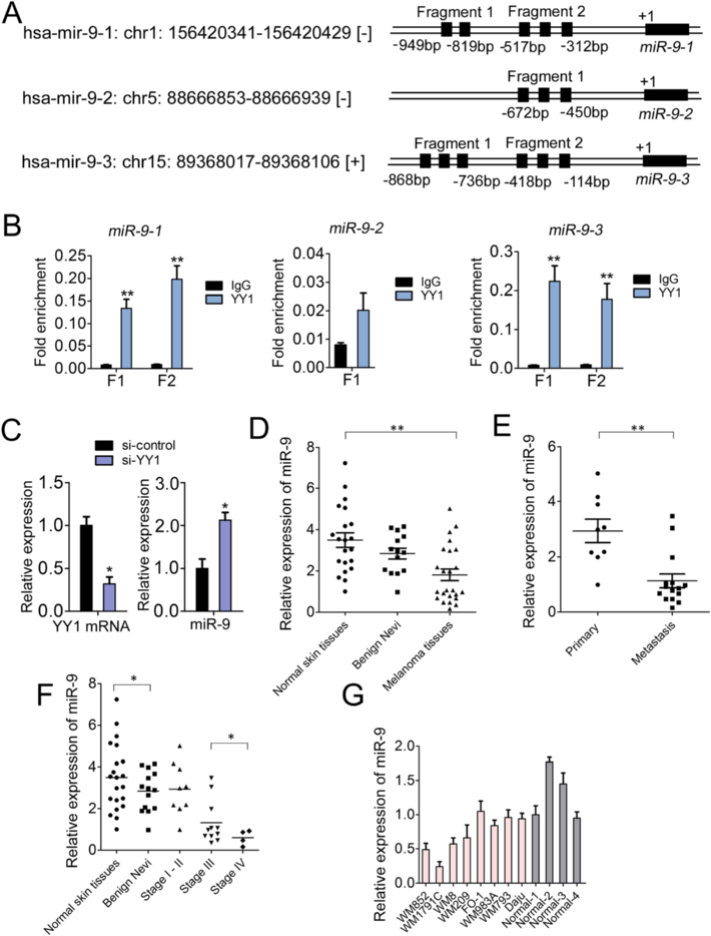
YY1 inhibites miR-9 expression in melanoma cells. (**a**) A representation of YY1 motifs scattered throughout the human miR-9-1, miR-9-2 and miR-9-3 loci. Fragment 1 and 2 represents the amplified fragments in subsequent ChIP-q-PCR analysis. (**b**) ChIP-q-PCR analysis of the YY1 hits on three miR-9 loci in WM1791C cells; (**c**) Q-PCR analysis of YY1 mRNA and miR-9 level in WM1791C cells treated with si-YY1 or si-control for 48 h; (**d**) Relative miR-9 expression levels in normal skin tissues, benign nevi and melanoma tissues; (**e**) The Statistical analysis of the association between miR-9 level and metastasis state (primary and metastasis); (**f**) The Statistical analysis of the association between miR-9 level and tumor stage (I, II, III and IV); (**g**) The relative level of miR-9 in melanoma cell lines (WM852, WM1791C, WM8, WM209, FO-1, WM983A, WM793 and Daju) relative to 4 normal tissue controls. For the quantitative results, the data are presented as the mean ± SEM, and the error bars represent the standard deviation obtained from three independent experiments. *, *p* < 0.05; **, *p* < 0.01

YY1 is a ubiquitously expressed transcription factor, and the YY1-miRNA axis, as NF-kB- YY1-miR-29 signaling axis in rhabdomyosarcoma [32, 33] and YY1-miR-1-Pax 7 axis in skeletal myogenesis [34], has been identified in different cellular processes. Considering the prevalence of YY1 binding sites in the genome, we speculate that many other miRNAs could come under regulation by YY1, forming numerous functional regulatory circuitries. Bioinformatics prediction revealed several putative YY1 binding sites scattered within ~1 kb upstream of the three miR-9 genomic loci (5 sites for *miR-9-1* locus, 3 sites for *miR-9-2* locus, 6 sites for *miR-9-3* locus; Fig. 3a). The binding of YY1 on these putative sites was examined by ChIP-q-PCR analysis and confirmed YY1 occupancy on the upstream of *miR-9-1* locus and *miR-9-3* locus, but not *miR-9-2* locus in WM1791C cells (Fig. 3b). Furthermore, to determine whether YY1 regulated the expression of miR-9, q-PCR was performed in WM1791C cells upon si-YY1 treatment (Fig. 3c). As expected, inhibition of YY1 increased miR-9 by ~2-fold, suggesting a transcriptional inhibition by YY1 on miR-9 in melanoma cells.

Given the regulatory roles of YY1 on miR-9 expression, we continued to detect whether the expression of miR-9 was altered in melanoma patients and cell lines. As shown in Fig. 3d, downregulation of miR-9 was seen in patients with melanoma compared to the normal tissue controls (*p* < 0.01). However, there was no statistical significance of miR-9 level between melanoma specimens and benign nevi. We further found that decreased miR-9 was associated with metastasis state (*p* < 0.01, primary vs. metastasis) (Fig. 3e) and different tumor stages stage (*p* < 0.05 Stage IV vs. Stage III) (Fig. 3f).

### Overexpression of miR-9 in melanoma cells inhibits cell proliferation, migration and invasion

**Fig. 4:**
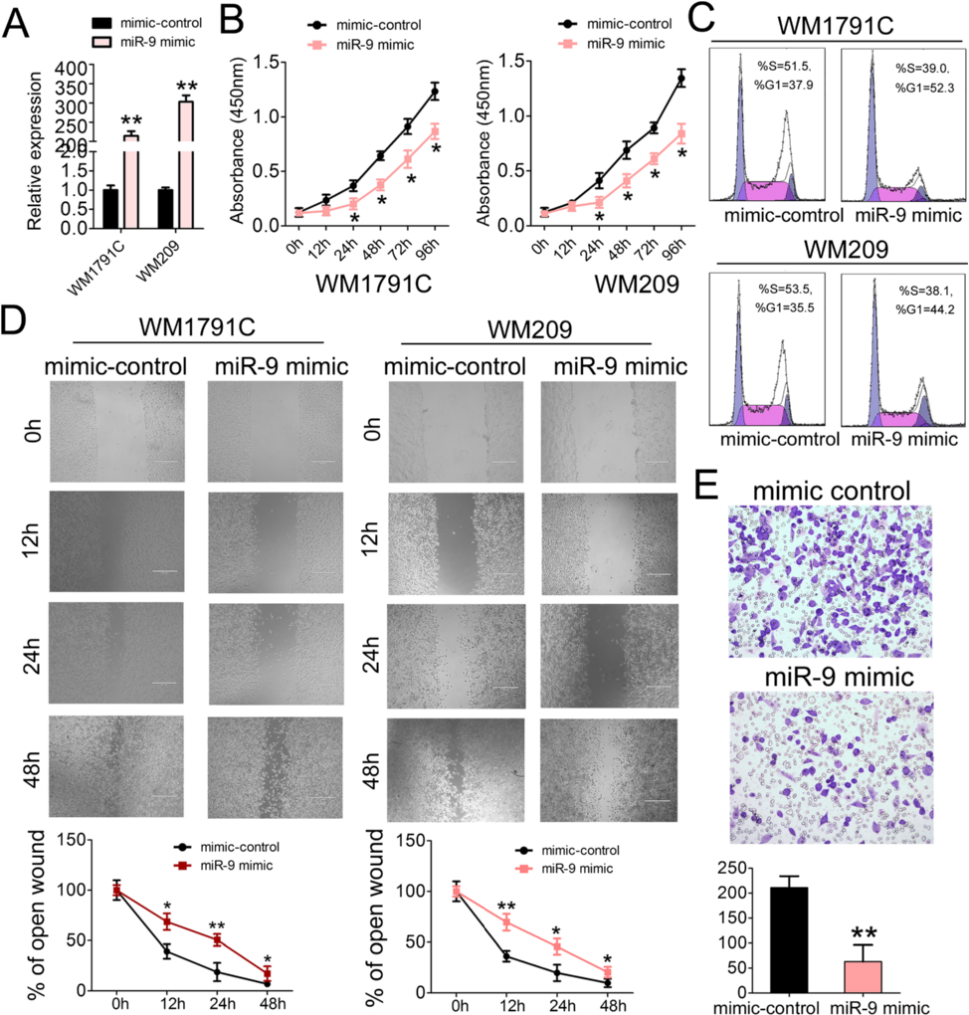
Overepression of miR-9 inhibits melanoma cell proliferation, migration and invasion. (**a**) miR-9 level were detected in WM1791C and WM209 cells after treatment with miR-9 mimic (25 nM) or mimic control (25 nM) by RT-qPCR; (**b**) Cell proliferation assay of WM1791C and WM209 cells after treatment with miR-9 mimic or mimic control by using CCK-8; (**c**) Cell cycle analysis by propidium iodide (PI) staining of WM1791C and WM209 cells after treatment with miR-9 mimic or mimic control; (**d**) Wound healing assays of WM1791C (the left panel) and WM209 (the right panel) cells after treatment with miR-9 mimic or mimic control, The relative ratio of wound closure per field was shown in the below; (**e**) Transwell analysis of WM1791C cells after treatment with miR-9 mimic or mimic control, The relative ratio of invasive cells per field was shown below. For the quantitative results, the data are presented as the mean ± SEM, and the error bars represent the standard deviation obtained from three independent experiments. *, *p* < 0.05; **, *p* < 0.01

To test the potential roles of miR-9 in melanoma carcinogenesis, we first detected the relative expression level of miR-9 in different melanoma cell lines and the results showed that miR-9 was significantly down-regulated in WM852, WM1791C, WM8 and WM209 (Fig. 3g). Of them, WM1791C and WM209 were selected to analyze the role of miR-9. RT-q-PCR was first used to measure the level of miR-9 after transfection and showed that the level of miR-9 was ~200-fold higher in miR-9 mimic-transfected WM1791C and WM209 cells (Fig. 4a). Accordingly, the CCK-8 proliferation assay in the same cells indicated that cell growth was suppressed after transfection with miR-9 mimic (Fig. 4b). Additionally, the cell cycle progression was also inhibited upon miR-9 mimic treatment (Fig. 4c). Furthermore, the wound healing assay showed that cell migration was inhibited in miR-9 mimic-transfected WM1791C and WM209 cells compare to the mimic control-transfected ones (Fig. 4d), suggesting the inhibitory effects of miR-9 on tumor cell migration. To detect whether miR-33b possesses the ability to inhibit cell invasion, transwell invasion assay was performed. As expected, there was significant reduction in cell invasiveness after miR-9 mimic transfection in WM1791C cell lines (Fig. 4e). Taken together, these results indicated the tumor suppressive roles of miR-9 in GC cells, which was opposite to its negative regulator YY1.

### YY1 ~ miR-9 ~ RYBP axis in melanoma cells

**Fig. 5:**
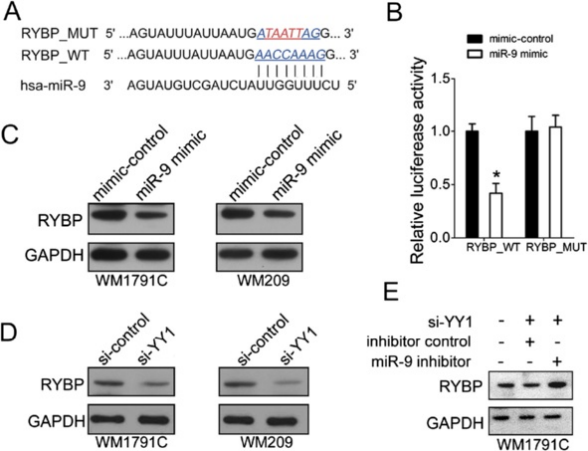
RYBP is repressed by miR-9 in melanoma cells. (**a**) A computer prediction of the conserved and mutated binding sites within the 3' UTR of human RYBP mRNAs for miR-9; (**b**) Relative luciferase activity of the indicated RYBP reporter constructs. Error bars represent the standard deviation obtained from three independent experiments; (**c**) Immunoblot analysis of RYBP in WM1791C and WM209 cells transfected with mimic control or miR-9 mimics; An unrelated protein GAPDH was used as the control. (**d**) Immunoblot analysis of RYBP in WM1791C and WM209 cells transfected with si-YY1 or si-control; An unrelated protein GAPDH was used as the control. (**e**) Immunoblot analysis of RYBP in WM1791C transfected with si-YY1, and miR-9 inhibitor or inhibitor control. An unrelated protein GAPDH was used as the control. For the quantitative results, the data are presented as the mean ± SEM, and the error bars represent the standard deviation obtained from three independent experiments. *, *p* < 0.05; **, *p* < 0.01

Biological role of miR-9 in melanoma cells promoted us to study its mechanism in carcinogenesis. To this aim, we started to search for the mRNA targets of miR-9. After alignment, we found a putative binding site of miR-9 in the 3′UTR of RYBP mRNA (Fig. 5a). To validate whether miR-9 targets RYBP, we first cloned the wild-type (RYBP_WT) or miR-9-binding site-mutant (RYBP_MUT) RYBP 3' UTRs (Fig. 5a) into a pMIR-reporter plasmid and co-transfected these constructs into 293 T cells with an miR-9 mimic or a mimic-control, respectively. Reporter assays in 293 T cells revealed that miR-9 significantly reduced the luciferase activities of wild-type RYBP reporters compared to the control (Fig. 5b). In contrast, the luciferase activities of the mutant reporters were not repressed by miR-9, indicating that the repression was dependent on miRNA binding (Fig. 5b). Moreover, immunoblotting assay was carried out in WM1791C and WM209 cells and showed that RYBP protein was about 2-fold lower in cells transfected with miR-9 mimics (Fig. 5c).

Based on above findings that YY1 negatively regulated miR-9 and miR-9 repressed RYBP expression, we speculated that the modification of YY1 level would ultimately changed RYBP expression. To test this hypothesis, we used si-YY1 to inhibit the endogenous YY1 activity in melanoma cells. As expected, YY1 knockdown resulted in an obvious decrease of RYBP level in both WM1791C and WM209 cells (Fig. 5d). Furthermore, to test whether the regulation of YY1 on RYBP expression is mediated by miR-9 in melanoma cells, we performed a “rescue” assay with the combination of si-YY1 and miR-9 inhibitor in WM1791C cells. As expected, si-YY1 treatment led to a 2-fold decrease in RYBP protein levels compared to the control (Fig. 5e, panel 1 and 2). Furthermore, the addition of miR-9 inhibitor following si-YY1 treatment resulted in the increase of RYBP protein compared to inhibitor controls (Fig. 5e, panel 2 and 3), which suggesting that the regulation of RYBP by YY is indeed mediated by miR-9.

**Fig. 6:**
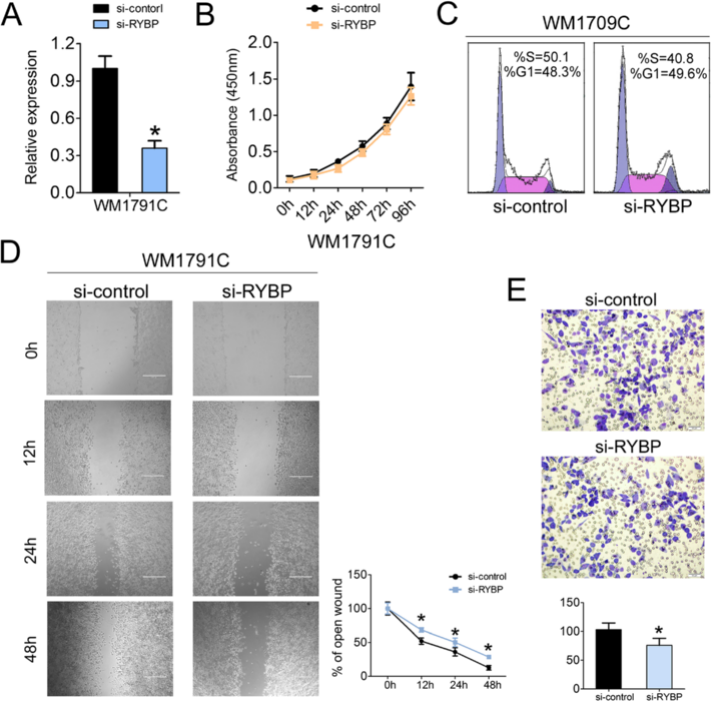
Knock-down of RYBP inhibits melanoma cell proliferation, migration and invasion. (**a**) RYBP level was detected in WM1791C cells after treatment with si-RYBP (25 nM) or si-control (25 nM) by RT-qPCR; (**b**) Cell proliferation assay of WM1791C cells after treatment with si-RYBP or si-control by using CCK-8; (**c**) Cell cycle analysis by propidium iodide (PI) staining of WM1791C cells after treatment with si-RYBP or si-control l; (**d**) Wound healing assays of WM1791C cells after treatment with si-RYBP or si-control, The relative ratio of wound closure per field was shown in the right; (**e**) Transwell analysis of WM1791C cells after treatment with si-RYBP or si-control The relative ratio of invasive cells per field was shown below. For the quantitative results, the data are presented as the mean ± SEM, and the error bars represent the standard deviation obtained from three independent experiments. *, *p* < 0.05; **, *p* < 0.01

The above findings made us to further investigate the potential involvement of RYBP in melanoma carcinogenesis. Firstly, the CCK-8 proliferation assay in the WM1791C cells showed that cell growth was a little suppressed after knock-down of RYBP by siRNAs (Fig. 6a, b). Accordingly, the cell cycle progression was also inhibited in RYBP silencing WM1791C cells. (Fig. 6c). Furthermore, the wound healing and transwell indicated that cell migration and invasion was respectively inhibited upon si-RYBP-transfected WM1791C cells compare to the si-control-transfected ones (Fig. 6d, e). Taken together, these results indicated that RYBP could regulate cell proliferation, migration and invasion of melanoma cells, further confirming the YY1 ~ miR-9 ~ RYBP axis in melanoma cells.

## Discussion

YY1 is a transcription factor with complex biological functions, including apoptosis, tumorigenesis, development and differentiation. Overexpression of YY1 in tumor tissues exerts different clinical behavior in different tumor types. Moreover, in many human cancer types, YY1 expression levels were found to be significantly elevated in the metastatic tumor compared to its primary counterpart, supporting the potential role of YY1 in cancer development [18, 20]. However, its involvement in melanoma tumorigenesis has not been well defined. In that regard, it would be interesting to test the biological and mechanistic significance of YY1 in melanoma. In general, our observations corroborated that YY1 was an oncogenic regulator for human melanoma. Therefore, its regulation in cancer along with the development of new therapeutic targets of YY1 may represent promising tools against melanoma therapy.

In the current study, RYBP, a PcG member was discovered to be a direct target of miR-9 in melanoma cells. PcG proteins are crucial for epigenetic inheritance of cell identity and are functionally conserved from Drosophila to human [35]. PcG proteins are also perturbed in a range of cancers, suggesting that they are critical for maintenance of normal cell identity [36–38]. In mammals, the PcG proteins are generally found in one of two protein complexes, the polycomb repressive complexes 1 or 2 (PRC1 or PRC2). Molecular characterization of the PRC complexes has revealed the combined activities of PRC1 and PRC2 in target gene recognization. Specifically, H3K27me3 placed by PRC2 is recognized by PRC1 complexes that contain chromobox (CBX) proteins, which represents a canonical model for PRC recruitment. Alternatively, the recent demonstration that variant PRC1 complexes (containing RYBP/YAF2 instead of CBX) bind to many target sites, albeit at lower levels, can be achieved in the absence of PRC2. Previous studies have identified RYBP as a transcription silencer in embryogenesis [39], skeletal myogenesis [34] and central nervous system development [40]. Our current study demonstrated a potential role of RYBP in melanoma tumorigenesis. Furthermore, RYBP and YY1 were found to co-occupy several target promoters/enhancers of YY1 to silence their expression, thereby a YY1 ~ miR-9 ~ RYBP feedback regulatory loop might be existed in melanoma cells.

## Conclusions

In line with the other studies, our results suggested YY1 as a transcriptional repressor of miR-9 and a promoter of melanoma growth and progression by modulating the miR-9 ~ RYBP axis.
